# Evolution at increased error rate leads to the coexistence of multiple adaptive pathways in an RNA virus

**DOI:** 10.1186/1471-2148-13-11

**Published:** 2013-01-16

**Authors:** Laura Cabanillas, María Arribas, Ester Lázaro

**Affiliations:** 1 Centro de Astrobiología (CSIC-INTA) Ctra de Ajalvir Km 4, Torrejón de Ardoz, Madrid, 28850, Spain

**Keywords:** RNA viruses, Interference, Beneficial mutations, Mutagenesis, 5-azacytidine, Epistasis, Polymorphisms

## Abstract

**Background:**

When beneficial mutations present in different genomes spread simultaneously in an asexual population, their fixation can be delayed due to competition among them. This interference among mutations is mainly determined by the rate of beneficial mutations, which in turn depends on the population size, the total error rate, and the degree of adaptation of the population. RNA viruses, with their large population sizes and high error rates, are good candidates to present a great extent of interference. To test this hypothesis, in the current study we have investigated whether competition among beneficial mutations was responsible for the prolonged presence of polymorphisms in the mutant spectrum of an RNA virus, the bacteriophage Qβ, evolved during a large number of generations in the presence of the mutagenic nucleoside analogue 5-azacytidine.

**Results:**

The analysis of the mutant spectra of bacteriophage Qβ populations evolved at artificially increased error rate shows a large number of polymorphic mutations, some of them with demonstrated selective value. Polymorphisms distributed into several evolutionary lines that can compete among them, making it difficult the emergence of a defined consensus sequence. The presence of accompanying deleterious mutations, the high degree of recurrence of the polymorphic mutations, and the occurrence of epistatic interactions generate a highly complex interference dynamics.

**Conclusions:**

Interference among beneficial mutations in bacteriophage Qβ evolved at increased error rate permits the coexistence of multiple adaptive pathways that can provide selective advantages by different molecular mechanisms. In this way, interference can be seen as a positive factor that allows the exploration of the different local maxima that exist in rugged fitness landscapes.

## Background

Beneficial mutations are the substrate upon which natural selection acts to drive adaptive evolution. For a beneficial mutation to fix in a population it is necessary that it survives genetic drift and that under the influence of selection displaces the rest of genomes. This means that any beneficial mutation remains as a polymorphism for a certain period of time, which in asexual populations lacking recombination is determined not only by its selective coefficient but also by the probability that other beneficial mutations are present at the same time in the population [1,2]. When beneficial mutations are scarce, they can be fixed before a secondary mutation arises, and adaptation takes place through the sequential fixation of mutations [3]. In contrast to this, when beneficial mutations are frequent, their fixation usually does not take place before one or more secondary beneficial mutations are generated. Genomes carrying the secondary mutations spread simultaneously with the genomes containing the primary one, delaying its fixation, and occasionally causing its elimination [4-10]. This competition among beneficial mutations is known as dynamics of interference. Whether a secondary mutation will displace a previously spreading one depends, among other factors, on the frequency reached by the first mutation when the second one arises, and on their relative selective values, which in the last term are determined by the distribution of beneficial mutation effects.

The frequency of beneficial mutations, and thus the intensity of interference, depends on several factors, among which some of the most relevant are the population size, the error rate, and the degree of adaptation of the population [11,12]. In large populations a high number of genomes replicate simultaneously, increasing the probability of generating beneficial mutations that can compete for fixation. In addition, the time necessary to fix any substitution is longer in large populations than in smaller ones, which also increases the opportunities for interference [13,14]. The increase of the error rate is associated with higher numbers of both deleterious and beneficial mutations per genome. In this situation, the fixation of beneficial mutations can be delayed not only by the coexistence of several lines carrying different mutations but also because at high error rate beneficial mutations arise in deleterious backgrounds, thus hampering the action of selection [14-16].

The dynamics of interference has been the focus of a number of theoretical and experimental studies. Theoretical studies establish different assumptions concerning the fitness effects of beneficial mutations and the genetic backgrounds where they can arise. The clonal interference model [13,14] assumes that most beneficial mutations have different selective values and always appear in the previously fixed background. The assumption that secondary mutations never appear in the genomes bearing a mutation that is in the process to fixation means that in this model competition takes place among genomes differing in a single beneficial mutation. By contrast, the multiple mutations model [17-19] assumes that beneficial mutations have a single characteristic selective value and can appear in any genetic background, including those containing beneficial mutations generated previously and still not fixed. In this way, competition can also occur among genomes differing in the number of beneficial mutations. Finally, the full interference model [20,21] tries to eliminate the limitations above by considering that beneficial mutations of different selective values can arise in any genetic background.

Each of the theoretical approaches predicts a particular dynamics of adaptation as a function of the population size and the error rate, and experimental studies have been designed to verify whether the behaviour of real populations of bacteria [11,22,23], yeast [17], or viruses [24] fit the theoretical predictions. Interference has also been studied at the genetic level by tracing specific mutations with particular labels [20,25], and by sequencing ensembles of individual genomes isolated from adapting populations [23,26-30]. This latter scenario allows analyzing the variation in the frequency and distribution of particular beneficial mutations along the adaptation process, which can shed much light about how interference affects the fate of beneficial mutations.

In this work we analyze the results of a previous evolution experiment carried out with a population of an RNA virus, the bacteriophage Qβ, evolved for a large number of generations in the presence of the mutagenic nucleoside analogue 5-azacytidine (AZC) [31] which increases the virus error rate [32]. The initial objective of the experiment was to characterize the possible mechanisms providing resistance to AZC in bacteriophage Qβ. It is important to note that during adaptation to mutagens, the same agent that acts as selective pressure also can interfere with the fixation of adaptive mutations, due to the increased number of errors generated in its presence. The analysis of the populations obtained at different points of the evolutionary process showed the fixation of two substitutions, A2187C that has a general beneficial effect, and A1746U with a selective advantage in the presence of AZC and a fitness cost in its absence [31]. In addition to these, six other mutations were detected as polymorphisms that, despite the demonstrated selective value of at least two of them, did not reach fixation after a large number of transfers in the presence of AZC. Since the conditions under which bacteriophage Qβ was propagated in this experiment (high mutation rate and large population sizes) favour the coexistence of multiple beneficial mutations, we thought that interference among beneficial mutations could be one of the reasons underlying the prolonged presence of polymorphisms. To further study whether this process operates during the evolution of bacteriophage Qβ at increased error rate, we have analyzed how polymorphic mutations are distributed in individual virus genomes isolated at different points of the evolutionary series. This approach has allowed us to identify several competing lines carrying different combinations of polymorphic mutations which differ in their fitness values and in their ability to fix when present in a simpler mutant spectrum. The presence of additional mutations accompanying the polymorphic ones, the high frequency of recurrent mutations, and the occurrence of epistatic interactions contribute to generate a highly complex dynamics that would likely require improved theoretical models to be successfully described.

## Methods

### Viruses and bacteria. Standard procedures for infection

Bacteriophage Qβ was routinely propagated by infecting log-phase cultures of *Escherichia coli*, strain Hfr (Hayes) in NB medium (8 g/l Nutrient Broth from Merck and 5 g/l NaCl). The virus was adapted to replication in liquid culture medium in our laboratory as described [32].

Infections in liquid medium were always carried out using fresh exponential phase *E. coli* cultures (with an optical density at 550 nm between 0.6 and 0.8) that were infected with the virus at the multiplicity of infection (moi) indicated in each experiment. After 2 h of incubation at 37°C with good aeration, cultures were treated with 1/20 vol of chloroform for 15 min at 37°C with shaking (300 rpm). Virus supernatants were harvested upon centrifugation at 13000 × g for 10 min and maintained at 4°C for short-term use (less than 15 days) or at -80°C for long-term storage. Virus titres were determined by plaque assay and expressed as the number of plaque forming units (pfu) per ml of the phage suspension.

Virus populations were used to obtain biological clones that correspond to lytic plaques obtained in semisolid agar. Virus clones were isolated by punching and removing the top and the bottom agar around well-separated lytic plaques. The agar containing the lytic plaque was transferred into an eppendorf tube with 1 ml of phage buffer (1 g/l gelatine, 0.05 M Tris–HCl, pH 7.5, and 0.01 M MgCl_2_) and 50 μl of chloroform, and incubated for 1 h at 28°C with shaking (300 rpm). After centrifugation at 13000 × g for 15 min to clarify the supernatant, the latter was stored over 25 μl of chloroform.

### Serial transfers of bacteriophage Qβ

**Figure 1:**
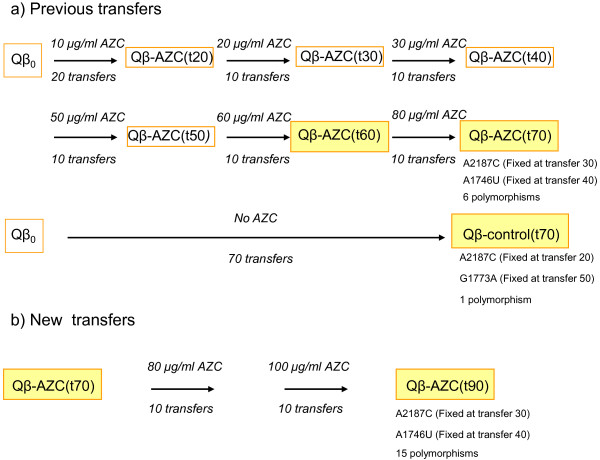
**Scheme showing the serial transfers experienced by bacteriophage Qβ. a)** Populations obtained in our previous work [31] that have also been used in the current work. **b)** Progression of the transfers series to obtain the new population Qβ-AZC(t90). The procedure describing how transfers were carried out is described in Methods. Populations were named Qβ-AZC(tx) or Qβ-control(tx) , where x indicates the number of transfer at which they were isolated. The mutations fixed and the number of polymorphic mutations at the end of each transfer series are also indicated. Boxes filled in yellow enclose populations where both the mutant spectrum and the consensus sequence have been analyzed. Non-filled boxes enclose populations analyzed only at the level of consensus sequence. Consensus sequences were analyzed from nucleotide 180 to 4180. Sequences from individual viruses spanned from nucleotide 1485 to 4028.

Prior transfers: A population of bacteriophage Qβ, previously adapted to replicate in our laboratory (population Qβ_0_), was used to infect two parallel cultures of *E. coli* in exponential phase at an initial moi = 1 pfu/cell in a volume of 10 ml either in the absence of AZC (population Qβ-control) or in the presence of a gradually increased AZC concentration (population Qβ-AZC) (Figure 1a). After 2 h of incubation at 37°C with good aeration, the virus supernatants were collected as described above, and 1 ml of each phage suspension was used to infect a fresh *E. coli* culture. Virus titres were determined each 10 transfers, which allowed us to estimate the number of viruses used to initiate each subsequent transfer. This procedure was repeated for a total of 70 transfers in both the control population and in the AZC-exposed population. Virus populations were isolated throughout the transfer series and the number of transfers experienced by each of them was indicated in brackets beside the name of the population (Figure 1a) [31].

New transfers: The population Qβ-AZC(t70) was subjected to 20 additional transfers, the first 10 in the presence of 80 μg/ml of AZC and the last 10 in the presence of 100 μg/ml of AZC (Figure 1b). Transfers were carried out as described above. The number of viruses used to initiate each subsequent transfer was always above 10^7^ pfu.

### RNA extraction, cDNA synthesis, PCR amplification and nucleotide sequencing

Virus RNA was prepared following standard procedures [31,32] from both complex populations, to determine the consensus sequence (from nucleotide 180 to nucleotide 4180), and biological clones, to determine individual virus sequences (from nucleotide 1485 to nucleotide 4028). Sequences were deposited in NCBI GenBank with accession numbers KC137648- KC137682.

RNAs were amplified by RT-PCR using Avian Myeloblastosis Virus RT (Promega) and Expand High Fidelity DNA polymerase (Roche). The cDNAs were purified with a Qiagen purification kit and subjected to cycle sequencing with Big Dye Chemistry (Applied Biosystems; Perkin Elmer). The following pairs of oligonucleotide primers were used for RT-PCR: P1 forward (5^′^CGAATCTTCCGACACGCATCC3^′^) with P1 reverse (5^′^AAACGGTAACACGCTTCTCCAG3^′^) to amplify from nucleotide position 150 to 1497; P2 forward (5^′^CTCAATCCGCGTGGGGTAAATCC3^′^) with P2 reverse (5^′^CAGAAAATCGGCAGTGACGCAACA3^′^) to amplify from nucleotide position 1407 to 2817; P3 forward (5^′^GTGCCATACCGTTTGACT3^′^) with P3 reverse (5^′^TCGTGCCCTGGAAGACC3^′^) to amplify from nucleotide position 2254 to 4095; and P4 forward (5^′^GCGGCAAGCACTACTATTCT3^′^) with P4 reverse (5^′^GATCCCCCTCTCACTCGT3^′^) to amplify from nucleotide position 3541 to 4195. Sequences were aligned with the consensus sequence of the wild type phage with Clustal W. Mutations relative to the consensus sequence were identified using the program BioEdit. Nucleotides were numbered according to the sequence of a cDNA of bacteriophage Qβ cloned in the plasmid pBR322 [33].

### Preparation of virus clones through directed mutagenesis of an expression vector of bacteriophage Qβ

The plasmid pBRT7Qβ, which contains the cDNA of the bacteriophage Qβ cloned in the plasmid pBR322 [33] was used to express the wild type virus (Qβ_wt_) and also mutant viruses containing specific substitutions. Mutagenesis was carried out using a QuickChange II Site-Directed Mutagenesis Kit from Stratagene. Primers 5^′^CTTAGACTCGTCTGAGGTGACTGTTTACGGAGACGA3^′^, 5^′^CCTCTTAGGGGTCCATCGAGTTGCGATTCTGCGG3^′^, 5^′^CCATCGATCAGCTTATCTGCAGGAGTAATCCTACGAAG3^′^, and their complementary were used to introduce the substitutions C3413G, G3945A, and U3989C respectively. The primers used to build the mutants containing A1746U, A2982G, and U3582C had been described previously [31]. The procedures to isolate the site-directed mutant viruses were as detailed in [31]. Each of the experiments carried out with viruses obtained upon expression of the mutagenized infectious clone was initiated with a single lytic plaque whose sequence had been previously analyzed to check the presence of the desired mutation.

### Determination of relative fitness values of bacteriophage Qβ mutant viruses

Growth rate values were used as a surrogate of fitness. To determine them, liquid cultures containing 10^8^ bacteria growing in exponential phase were inoculated with 10^4^ pfu of the site-directed mutant indicated in a final volume of 1 ml. After two hours at 37°C with good aeration, the virus supernatants were collected as described above and titrated to estimate the virus yield. Preliminary assays showed that the virus used as reference (the wild type virus, Qβ_wt_, obtained upon expression of the infectious clone of bacteriophage Qβ described in the previous section) grew exponentially during this time interval. Growth rate determinations for each virus were carried out in triplicate in an assay which always included the reference virus. Absolute fitness for a given mutant was calculated as the change in log_2_ of the virus titre, and relative fitness was defined as the ratio between the absolute fitness of the mutant assayed and the absolute fitness of the reference virus.

### Competition between bacteriophage Qβ virus clones

Competitions between bacteriophage Qβ virus clones (Qβ_wt_ and the site-directed mutants indicated in each experiment) were carried out by mixing equal amounts of each competitor virus (10^6^ or 10^7^ pfu from each of them) which were used to infect 10^9^ bacteria in a final volume of 10 ml. Each new transfer was initiated with 1 ml of a dilution of the previous virus supernatant to maintain a moi about 0.01. All the competitions were carried out in duplicate. Evolution took place either in the presence (60 μg/ml) or in the absence of AZC. The population obtained after the number of transfers indicated was sequenced to determine whether one of the viruses had become dominant.

## Results

### Previous results

In our previous work [31] a population of bacteriophage Qβ was evolved during 70 transfers under two parallel transmission regimes that differed in the presence of the mutagenic nucleoside analogue AZC. In this way we obtained the population Qβ-control(t70) evolved at the standard error rate of the virus, and the population Qβ-AZC(t70) evolved at artificially increased error rate (Figure 1a). These populations had in common the fixation of substitution A2187C, and differed in the presence of substitutions A1746U, which was only fixed in population Qβ-AZC(t70), and substitution G1773A, which was exclusive of population Qβ-control(t70) (Figure 1a). Population Qβ-AZC(t70) showed a statistically significant higher mutation frequency in the mutant spectrum than population Qβ-control(t70) (3 × 10^-3^ versus 2.5 × 10^-4^ substitutions per nucleotide). In addition, 6 polymorphic mutations (U1520C in the coat protein gene, and A2982G, C3413G+U, U3582C, G3945A, and U3989C in the replicase gene) were detected in population Qβ-AZC(t70). In contrast to this result, population Qβ-control(t70) showed no polymorphisms in its consensus sequence, and only one substitution was represented at a frequency of 0.2 when the mutant spectrum was analyzed through sequencing of 10 biological clones.

Competition experiments between the wild type virus and two single-directed mutants containing the replicase polymorphic substitutions A2982G and U3582C showed that both could fix in only five transfers in the presence of AZC, which clearly demonstrates that they have a selective advantage under this condition, and can fix when the complexity of the mutant spectrum is reduced. These results motivated us to investigate how the complexity of the mutant spectra generated at increased error rate interferes with the fixation of beneficial mutations.

### Extended evolution of population Qβ-AZC(t70) in the presence of AZC leads to a further increase in the number of polymorphims

Population Qβ-AZC(t70) described in the previous section was subjected to 20 additional transfers, the first 10 in the presence of 80 μg/ml of AZC and the last 10 in the presence of 100 μg/ml of AZC (Figure 1b). In this way we obtained the population Qβ-AZC(t90), evolved during 90 transfers in the presence of AZC. The analysis of the consensus sequence of this population revealed that none of the polymorphic mutations previously identified was fixed at transfer number 90. Moreover, the analysis of 15 biological clones isolated from population Qβ-AZC(t90) revealed the presence of 9 additional substitutions that were represented at high frequency (≥ 0.2) in the mutant spectrum (Table 1).

**Table 1:** Distribution of polymorphic mutations in individual viruses isolated from bacteriophage Qβ populations evolved in the presence of AZC

**Nt**^**1**^	**1520**	**1604**	**2059**	**2277**	**2378**	**2384**	**2471**	**2982**	**3413**	**3545**	**3582**	**3879**	**3945**	**3989**	**4006**
**Wt**^**2**^	**U**	**C**	**C**	**A**	**C**	**C**	**C**	**A**	**C**	**C**	**U**	**C**	**G**	**U**	**A**
Population Qβ-AZC(t60)^3^															
C_1_	C	G	.	.	.	.	.	G	U	.	.	.	A	C	.
C_2_	C	.	.	.	.	.	.	G	.	.	.	.	A	C	.
C_3_	C	.	.	.	.	.	.	G	.	.	.	.	A	C	.
C_4_	C	G	.	.	.	.	G	G	.	.	.	.	A	C	.
C_5_	.	.	.	.	.	.	.	.	.	.	.	.	.	.	.
C_6_	.	.	.	.	.	.	.	.	G	.	.	.	.	.	G
C_7_	.	.	.	G	G	.	.	.	.	.	.	.	.	.	.
C_8_	.	.	.	.	.	G	.	.	.	.	.	.	.	.	.
C_9_	.	.	.	.	.	G	G	.	.	.	.	.	.	.	.
C_10_	.	.	.	.	.	.	.	.	.	.	.	.	.	C	.
Population Qβ-AZC(t70)^3^															
C_1_	C	.	.	.	.	.	.	G	.	.	.	.	A	C	.
C_2_	C	.	G	.	.	.	.	G	.	.	.	.	A	C	.
C_3_	C	.	.	.	.	G	.	G	G	.	.	.	A	C	.
C_4_	.	.	.	.	.	.	.	.	.	.	C	.	.	.	.
C_5_	.	.	.	.	.	.	G	.	.	.	C	.	.	.	.
C_6_	.	.	.	.	.	.	.	.	G	.	C	.	A	C	.
C_7_	.	.	G	.	.	G	.	.	G	.	.	.	.	.	.
C_8_	.	.	.	.	.	.	.	.	G	.	.	.	.	.	.
C_9_	.	.	.	.	.	.	.	.	.	.	.	.	.	.	.
C_10_	.	.	.	.	.	.	G	.	.	.	.	G	.	.	.
Population Qβ-AZC(t90)^3^															
C_1_	C	.	.	.	.	.	.	G	.	.	.	.	A	C	.
C_2_	C	G	.	.	.	.	G	G	.	A	.	.	A	C	G
C_3_	C	.	G	G	G	.	.	G	.	.	.	.	A	C	.
C_4_	C	G	.	.	.	.	.	G	.	A	.	.	A	C	G
C_5_	C	.	G	.	.	U	.	G	.	.	.	.	A	C	.
C_6_	C	.	G	G	.	.	G	G	.	.	.	.	A	C	.
C_7_	C	.	.	G	.	.	.	G	.	A	.	G	A	C	.
C_8_	C	G	G	.	.	.	G	G	G	A	.	.	A	C	G
C_9_	.	.	.	.	G	.	.	.	.	.	C	G	.	.	.
C_10_	.	.	.	.	G	.	.	.	.	.	C	.	.	.	G
C_11_	.	.	G	G	.	U	.	.	G	.	C	.	A	C	.
C_12_	.	.	.	G	.	G	.	.	.	.	.	.	.	.	.
C_13_	.	G	.	.	G	U	.	.	.	.	.	.	.	.	.
C_14_	C	.	.	G	.	G	.	.	.	.	.	.	A	.	.
C_15_	.	.	.	.	.	.	.	.	G	.	.	G	A	C	.

^1^Nucleotide positions where polymorphisms were detected.

^2^Nucleotides present at the positions indicated in the wild type virus.

^3^Nucleotides present at the positions indicated in the virus clones (indicated as C_x_, where x is a number arbitrarily assigned) isolated from that populations. The region sequenced comprises from nucleotide 1485 to nucleotide 4028.

To study how polymorphic mutations distribute in individual virus genomes along the evolutionary series carried out in the presence of AZC, we also analyzed the mutant spectrum of two previous populations [Qβ-AZC(t60) and Qβ-AZC(t70), see Figure 1. We isolated 10 biological clones from each population and sequenced the genomic region where polymorphic substitutions had been identified in our prior work [31]. We sequenced biological clones instead of molecular clones to ensure the analysis of viable viruses and to identify possible associations among mutations in the same genome. The whole list of polymorphisms, as well as their distribution in the virus genomes analyzed is shown in Table 1. All the genomes listed in Table 1 also carry substitutions A2187C, which was fixed a transfer number 30, and A1746U, which was fixed at transfer number 40, together with a number of additional mutations that were exclusive of each of them (see Additional file 1). All sequences were submitted to NCBI GenBank. Their accession numbers are KC137673-KC137682 (virus clones from population Qβ-AZC(t60), KC137663-KC137672 (virus clones from population Qβ-AZC(t70), and KC137648-KC137662 (virus clones from population Qβ-AZC(t90)).

Another criterion to identify polymorphic mutations is their presence as double bands, consisting of a mixture of the mutated and the wild nucleotides, in the chromatograms of the consensus sequences of the corresponding virus populations. In good agreement with this expectation, all substitutions represented at a frequency ≥ 0.2 in the mutant spectrum of a given population appeared also as double peaks in the chromatogram of the consensus sequence of the same population. This approach was also used to identify the transfer number at which polymorphisms could be first detected. To this end we examined the chromatograms corresponding to the consensus sequences of populations Qβ-AZC(t20), Qβ-AZC(t30), Qβ-AZC(t40), and Qβ-AZC(t50). We observed that some polymorphisms could be detected as double bands in the chromatograms at transfers as early as 30, whereas others could not be detected until transfer number 90 (Table 2). Substitution U3989C which in our previous work [31] was identified as a polymorphism at transfer number 40 together with A3945G, was actually already present as a double band at transfer number 30.

**Table 2:** Substitutions that remain as polymorphisms at transfer number 90 in the bacteriophage Qβ evolved in the presence of AZC

**Substitution**	**Gene**^**1**^	**change**^**2**^	**First detected**^**3**^
U1520C	Coat	Syn	50
C1604G	Coat	Syn	60
C2059G	Read-through	T/S	60
A2277G	Read-through	K/E	90
C2378G	Replicase	N/K	90
C2384(G+U)	Replicase	Syn	60
C2471G	Replicase	Syn	30
A2982G	Replicase	T/A	50
C3413(U+G)	Replicase	Syn	50
C3545A	Replicase	Syn	70
U3582C	Replicase	Y/H	50
C3879G	Replicase	L/V	70
G3945A	Replicase	G/S	40
U3989C	Replicase	Syn	30
A4006G	Replicase	K/R	60

^1^Location of each substitution in the genome of the bacteriophage Qβ.

^2^Type of change produced by each nucleotide substitution. In the case of non synonymous changes, the amino acid substitution is indicated.

^3^Transfer number at which each substitution was first detected as a double band in the chromatogram corresponding to the consensus sequence of that population. The genomic region analyzed comprised from nucleotide 180 to 4180.

The location of polymorphic substitutions in the genome of bacteriophage Qβ shows a clear preference for the replicase gene (Table 2). However, we must take into account that the lysis gene and the starting of the coat protein gene were only analyzed at the level of consensus sequences. Although we did not observe any double band in the chromatograms corresponding to these regions, we cannot exclude that some polymorphism could have been detected upon the analysis of the mutant spectrum.

### Polymorphic mutations differ in their effects on fitness and in their ability to fix when present in a simple mutant spectrum

An explanation for the abundance of polymorphic mutations is that they have a selective advantage that permits them to increase their frequency thanks to the action of selection. To check this assumption we determined separately the ratio ds/dn for the polymorphic and non polymorphic mutations present in each population analyzed (Table 3). When mutations repeated in several genomes of the same population were counted only once, we found that the average ratio ds/dn was larger than 1 in both cases, rejecting the action of positive selection. However, the average ds/dn value obtained for the polymorphic mutations was significantly lower (*p* < 0.05, Student’s *t* test for the difference of means) than that estimated for the non polymorphic substitutions. A similar analysis carried out counting the repeated mutations the number of times that they appear in each population led to different results. In this case the average ds/dn obtained for the polymorphic substitutions was reduced to a value close to 1, whereas the average for the non polymorphic was kept almost unaltered (*p* > 0.05, Student’s *t* test for the difference of means) (Table 3). These findings suggest that positive selection acts at a higher extent in the substitutions represented as polymorphisms than in the non polymorphic.

**Table 3:** Ratio ds/dn for the nucleotide substitutions found in the bacteriophage Qβ populations evolved in the presence of AZC

	**ds/dn**^**1**^			
	**Repeated mutations counted once**		**Repeated mutations counted all the times they appear**	
**Population**	**Non polymorphic substitutions**^**2**^	**Polymorphic substitutions**^**2**^	**Non polymorphic Substitutions**^**2**^	**Polymorphic Substitutions**^**2**^
Qβ-AZC(t60)	5.7	4.0	5.3	1.7
Qβ-AZC(t70)	5.7	2.7	6.9	1.5
Qβ-AZC(t90)	7.2	3.2	7.1	1.6
Average^3^	6.2 ± 0.9	3.3 ± 0.7	6.4 ± 1.0	1.6 ± 0.1

^1^The value ds/dn indicates the ratio of synonymous to non synonymous substitutions corrected by the potential number of synonymous and non synonymous positions. The genomic region analyzed comprised from nucleotide 1485 to nucleotide 4028, excluding the non coding region (from nt 2331 to nt 2351). The number of synonymous and non synonymous positions was evaluated using the program SNAP [34,35].

^2^Polymorphic substitutions (those shown in Table 1 together with the fixed substitutions A1746U and A2187C) and non polymorphic substitutions (those shown in Additional file 1) were analyzed separately. All these mutations were placed in coding regions.

^3^Average value of the ratio ds/dn for the 3 populations analyzed. The difference between the average values obtained for non polymorphic and polymorphic substitutions was significant (*p* < 0.01 in both analysis, Student’s *t* test for the difference of means).

There are, however, two particular situations in which deleterious or neutral mutations can also reach high frequencies. The first one is the occurrence of population bottlenecks, a circumstance that reduces the genetic diversity, leading to the fixation of mutations independently of their selective value [2,36-38]. The second one is hitchhiking with beneficial mutations. Since the bacteriophage Qβ populations analyzed in this work were propagated using large population sizes (above 10^7^ pfu, see Methods), we can discard the first possibility. We neither found clear evidences of hitchhiking for most of the substitutions analyzed. If a mutation had reached high frequency because of its presence in the same genome where a beneficial mutation is generated, both mutations should appear always linked and should have been first detected at the same transfer number. The only two substitutions that meet these two requirements were U1520C and A2982G (see Tables 1 and 2). Since A2982G is beneficial in the presence of AZC [31], the only mutation that seems to have reached high frequency in our experiment because of hitchhiking is U1520C. However, as we will explain in the discussion, we cannot discard that some neutral or deleterious mutations could have achieved high frequency due to hitchhiking, even in the absence of a perfect association with a beneficial mutation.

To ascertain whether some of the polymorphic substitutions shown in Table 1 have selective advantages we prepared single site-directed mutants containing A1746U and the polymorphic substitutions that had been identified in population Qβ-AZC(t70) (Qβ_A1746U,_ Qβ_A2982G_, Qβ_U3582C_, Qβ_C3413G_, Qβ_G3945A_, and Qβ_U3989C_), and calculated their relative fitness values using the wild type virus, Qβ_wt_, as reference (see Methods). We observed that, with the only exception of Qβ_G3945A_, all the mutants assayed had lower fitness than the virus Qβ_wt_ in the absence of AZC (Table 4). In contrast to this, mutant viruses Qβ_A2982G_, Qβ_U3582C_, and Qβ_G3945A_ had higher fitness than the virus Qβ_wt_ in the presence of AZC (Table 4). To check whether the mutations contained in the site-directed mutants assayed can fix when present in a simple mutant spectrum, we carried out competition experiments of these viruses with the virus Qβ_wt_. After a number of transfers either in the presence or in the absence of AZC we determined the consensus sequences of the new populations generated to check the status of the substitutions analyzed (Table 4). In good agreement with their relative fitness values, mutants Qβ_A2982G_, Qβ_U3582C_, and Qβ_G3945A_ fixed in the presence of AZC, whereas only Qβ_G3945A_ fixed in the absence of AZC. These results indicate that substitution G3945A has a general beneficial fitness effect that is independent of the presence of AZC. Taking into account that A2982G and U3582C were fixed in only five transfers in the presence of AZC, whereas G3945A needed 15 transfers (Table 4), we can state that the last substitution has lower selective advantage under this condition than the first ones. Substitution C3413G remained as a polymorphism after 15 transfers in both the presence and the absence of AZC, which agrees with their fitness values close to neutrality. There is a disagreement between the relative fitness value of Qβ_A1746U_ in the presence of AZC (lower than 1) and the fact that this virus was able to displace the virus Qβ_wt_ when both competed under this condition. This discrepancy suggests that the growth rate of a virus cannot always be a good predictor of its behaviour when it propagates in the presence of competitor genomes.

**Table 4:** Relative fitness of bacteriophage Qβ site-directed mutants

**Site-directed mutant**	**Relative fitness**^**1**^	**Relative fitness**^**1**^	**Dominant virus in competition with Qβ**_**wt**_^**2**^	
	**+AZC**	**-AZC**	**+AZC**	**-AZC**
Qβ_A1746U_	0.71 ± 0.02^*^	0.58 ± 0.03^*^	Qβ_A1746U_	Qβ_wt_
Qβ_A2982G_	1.24 ± 0.08^*^	0.89 ± 0.05^*^	Qβ_A2982G_	Qβ_wt_
Qβ_U3582C_	1.31 ± 0.03^*^	0.89 ± 0.06^*^	Qβ_U3582C_	Qβ_wt_
Qβ_C3413G_^3^	0.95 ± 0.04^*^	0.92 ± 0.07^*^	Qβ_C3413G +_ Qβ_wt_ (transfer 15)	Qβ_C3413G +_ Qβ_wt_ (transfer15)
Qβ_G3945A_^3^	1.1 ± 0.06^*^	1.02 ± 0.05	Qβ_G3945A_ (transfer15)	Qβ_G3945A_ (transfer15)
Qβ_U3989C_	0.79 ± 0.04^*^	0.88 ± 0.05^*^	Qβ_wt_	Qβ_wt_

^1^Relative fitness was evaluated with respect to the virus Qβ_wt_ either in the absence or the presence of AZC (60 μg/ml). The absolute fitness value estimated for Qβ_wt_ was 15.42 ± 0.50 in the absence of AZC and 9.68 ± 0.22 in its presence. The asterisk indicates that the difference between the fitness values of the site-directed mutant and the virus Qβ_wt_ was significant (*p* value < 0.05, Student’s *t* test for the difference of means).

^2^Competition between the site-directed mutants indicated and the virus Qβ_wt_ was carried out as described in Methods during 5 transfers either in the absence or the presence of AZC (60 μg/ml).

^3^Since no virus became dominant at transfer number 5, competitions carried out with viruses Qβ_C3413G_ and Qβ_G3945A_ were extended until transfer number 15, yielding the results shown in the table.

**Figure 2:**
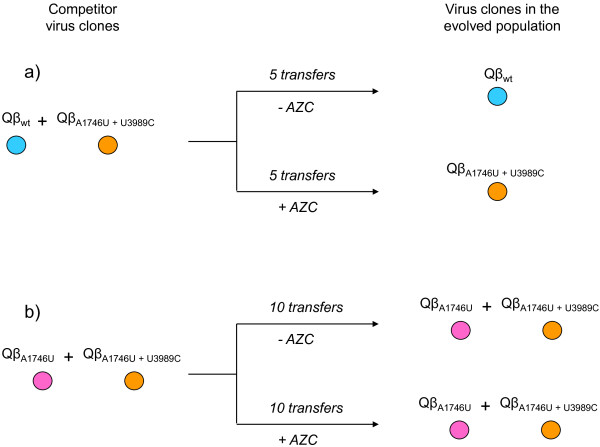
**Competition between different bacteriophage Qβ virus clones. a)** Qβ_wt_ and Qβ_A1746U+U3989C_. **b)** Qβ_A1746U_ and Qβ_A1746U+U3989C_. The experiment was carried out as described in Methods. The populations obtained after the number of transfers indicated were sequenced to determine whether one of the competitor viruses had become dominant.

The results showing that substitution U3989C was deleterious (Table 4) were puzzling since this substitution reached high frequency in the absence of apparent hitchhiking with any other substitution (Table 1). A possibility is that substitution U3989C is a compensatory mutation that raised high frequency because it reduces the fitness cost of another mutation previously selected. U3989C was generated in the genomic context of A2187C and A1746U. Since A1746U had a fitness cost in the absence of AZC (Table 4) [31], this substitution was the most probable candidate to be compensated by U3989C. To investigate this point we built a site-directed double mutant containing both substitutions A1746U and U3989C (Qβ_A1746U+U3989C_), and determined its relative fitness in the presence and in the absence of AZC. The value obtained in the presence of AZC (0.89 ± 0.2) was significantly higher than those obtained for the single mutants Qβ_A1746U_ and Qβ_U3989C_ (*p* < 0.05, Student’s *t* test for the difference of means). However, the relative fitness value obtained in the absence of AZC (0.80 ± 0.1) was only significantly higher that that obtained for the single mutant Qβ_A1746U._ Competition experiments of the double mutant with the virus Qβ_wt_ showed that after five transfers, the wild virus again dominated in the absence of AZC, indicating that under this condition substitution U3989C was not able to compensate the fitness cost of A1746U (Figure 2a). In contrast to this, the double mutant was selected in the presence of AZC, showing that in this case the combined effect of both substitutions was beneficial. Only with this result we cannot distinguish whether the advantage provided by A1746U in the presence of AZC is strong enough to cause the hitchhiking of U3989C or the double mutant Qβ_A1746U+U3989C_ is more advantageous than the single mutant Qβ_A1746U_. To test the last possibility we performed another competition experiment between the double mutant Qβ_A1746U+U3989C_ and the single mutant Qβ_A1746U_ (Figure 2b). After 10 transfers in both the presence and the absence of AZC, both viruses remained in the population. The result indicates that the effect of substitution U3989C was less deleterious in populations where substitution A1746U was previously fixed than in the mutational context of the wild type virus.

### Some polymorphic substitutions appear in multiple mutational contexts whereas other ones cannot coexist in the same genome

The analysis of the virus sequences displayed in Table 1 shows that most polymorphic substitutions are found in multiple mutational contexts, which can be indicative of a high frequency of recurrent mutations in bacteriophage Qβ. In contrast to this result, substitutions A2982G and U3582C were never located in the same genome. This finding is even more surprising if we take into account that A2982G and U3582C have selective advantages in the presence of AZC and, hence, their combination in the same genome could lead to a better adapted virus. Therefore, we were interested in analyzing how the behaviour of the double mutant would be. To this end, we introduced both mutations in the infectious clone of bacteriophage Qβ (see Methods), and tried to recover the viruses expressed. Sequencing of the plasmid DNA extracted from five *E. coli* transformed colonies showed that the site-directed mutagenesis had been successful. However, using the same conditions that let us obtain viruses when transformation took place with the infectious clone containing any of the two single mutations, we were not able to recover virus from any of the bacteria transformed with the infectious clone containing both substitutions. This result strongly suggests that the presence of both A2982G and U3582C in the same genome either is lethal for the virus or highly deleterious, a conclusion that agrees with the absence of any mutant containing both substitutions in the mutant spectrum of the populations analyzed in this work (Table 1), and also with the fitness costs that both substitutions have in the absence of AZC (Table 4) [31].

### Bacteriophage Qβ populations evolved in the presence of AZC are composed by genomes that can be grouped into multiple evolutionary lines

The observation that the combination in the same genome of substitutions A2982G and U3582C, both with clear selective advantages in the presence of AZC, is lethal or highly deleterious, and, hence, they are never associated in the same genome suggests that during the evolution of bacteriophage Qβ under mutagenic conditions, at least two evolutionary lines that evolve independently are generated. The first line would comprise virus clones C_1_ to C_4_ in population Qβ-AZC(t60), C_1_ to C_3_ in population Qβ-AZC(t70), and C_1_ to C_8_ in population Qβ-AZC(t90) (Table 1). Genomes from these viruses have in common the presence of substitution A2982G, accompanied by U1520C, G3945A, and U3989C. The second line would contain the virus clones C_4_ to C_6_ in population Qβ-AZC(t70), and C_9_ to C_11_ in population Qβ-AZC(t90), all carrying substitution U3582C (Table 1). Finally, a third line could be established containing the remaining genomes, which lack a clear set of polymorphic mutations in common.

**Figure 3:**
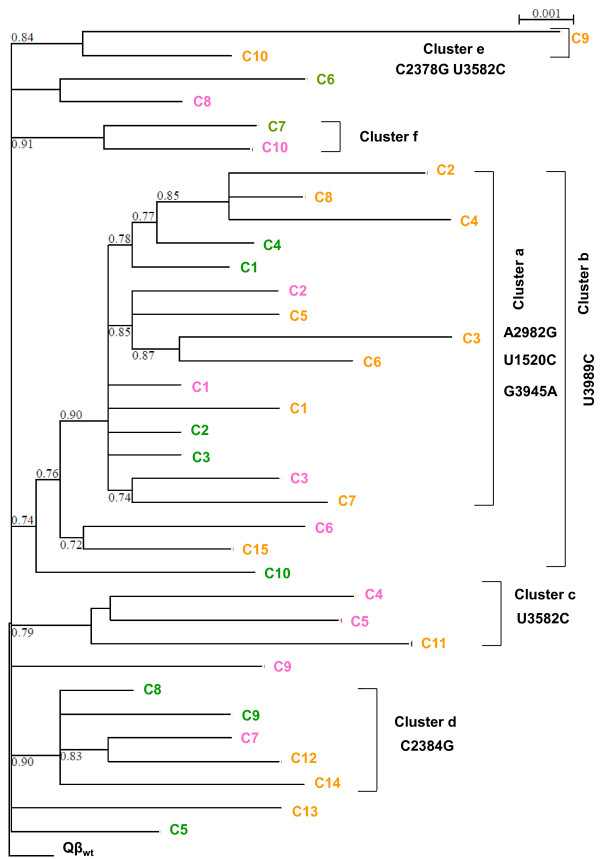
**Phylogenetic analysis of the virus genomes isolated from bacteriophague Qβ populations evolved in the presence of AZC.** Different colours are used to distinguish the genomes from each population included in the analysis: green for Qβ-AZC(t60), pink for Qβ-AZC(t70), and orange for Qβ-AZC(t90). Virus genomes corresponded to those shown in Table 1 and Additional file 1, and are identified using the same notation. The tree was derived by maximum likelihood methods (PhyML, program seaview 4) [39] using the sequence of the wild type virus to root the tree. Numbers at each node represent the bootstrap value (carried out with 100 replicates). Clusters described in the main text are highlighted, and the mutations common to all the genomes included in each one are also indicated.

A phylogenetic analysis carried out with the 35 genomes analyzed in this work (Figure 3) shows that all the genomes that we had previously included in line 1 (those carrying substitution A2982G, together with U1520C, G3945A, and U3989C) group into a cluster with a high bootstrap value (cluster a). This cluster is a part of another one (cluster b) that contains some additional genomes carrying substitution U3989C. The rest of genomes groups into 4 independent clusters differing in their polymorphic substitutions (Figure 3). It is remarkable that whereas all the genomes containing substitution A2982G are grouped into the same cluster, genomes containing substitution U3582C are placed in different ones, suggesting that this substitution has appeared repeatedly in different evolutionary lines.

Taking into account the transfer number at which each polymorphism was first detected (Table 2), a feasible sequence of events for the generation of the different lines can be established. Genomes containing A2187C (fixed at transfer number 30) and A1746U (fixed at transfer number 40) evolved through the acquisition of additional substitutions that can become represented at high frequency. One of the mutations appearing first was U3989C, which spreaded giving rise to the genomes included in clusters a and b (Figure 3). The remaining genomes continued replicating, and in this process they acquired some of the other polymorphic mutations listed in Table 1. Genomes belonging to different lines can compete among them, delaying the fixation of their mutations and allowing for the prolonged presence of a high number of polymorphisms. Moreover, genomes within each line can differ in additional polymorphic substitutions (see Table 1), and also in mutations that are exclusive of each of them (Additional file 1), which can modify their selective advantages adding further complexity to the competition process.

### The presence in different genomes of mutations having a selective advantage in the presence of AZC interferes with their fixation

To demonstrate whether the coexistence of multiple evolutionary lines carrying different mutations with selective advantages in the presence of AZC interferes with their fixation, we compared the results of competition experiments carried out between different site-directed mutants (Qβ_A1746U_, Qβ_A2982G,_ and Qβ_U3582C_) and between each one of the mutants and the virus Qβ_wt_ (Table 5).

**Table 5:** Competition among different site-directed mutants of bacteriophage Qβ in the presence of AZC

**Competitor viruses**	**Viruses present in the final population**^**1**^	
	**Transfer number 5**	**Transfer number 20**
Qβ_wt_ + Qβ_A1746U_	Qβ_A1746U_	ND^2^
Qβ_wt_ + Qβ_A2982G_	Qβ_A2982G_	ND
Qβ_wt_ + Qβ_U3582C_	Qβ_U3582C_	ND
Qβ_A1746U_ + Qβ_A2982G_	Qβ_A1746U_	ND
Qβ_A1746U_ + Qβ_U3582C_	Qβ_A1746U_	ND
Qβ_A2982G_ + Qβ_U3582C_	Qβ_A2982G_ +	Qβ_A2982G_ +
	Qβ_U3582C_	Qβ_U3582C_
Qβ_A1746U_ + Qβ_A1746U + A2982G_	Qβ_A1746U + A2982G_	ND
Qβ_A1746U_ + Qβ_A1746U + U3582C_	Qβ_A1746U + U3582C_	ND
Qβ_A1746U + A2982G_ + Qβ_A1746U + U3582C_	Qβ_A1746U + A2982G_ +	Qβ_A1746U + A2982G_ + Qβ_A1746U + U3582C_
	Qβ_A1746U + U3582C_	
Qβ_A1746U_ + Qβ_A1746U + A2982G_ + Qβ_A1746U + U3582C_	(Qβ_A1746U_)^3^ +	(Qβ_A1746U_)^3^ +
	Qβ_A1746U + A2982G_ +	Qβ_A1746U + A2982G_ +Qβ_A1746U + U3582C_
	Qβ_A1746U + U3582C_	

^1^Competitions were carried out as described in Methods. The viruses present in the populations obtained at the transfer indicated were identified through the determination of the consensus sequences at the nucleotide positions that distinguish the competitor viruses.

^2^ND means not determined.

^3^In this case, the determination of the consensus sequence do not allow distinguishing whether Qβ_A1746U_ is present in the populations analyzed or not. We show this virus in brackets to indicate this uncertainty.

Our results show that the virus Qβ_A1746U_ became dominant after 5 transfers when it competed with either Qβ_A2982G_ or Qβ_U3582C_, as it happened when the competitor was the wild type virus (Table 5). In contrast to this result, competition between the site-directed mutants Qβ_A2982G_ and Qβ_U3582C_ rendered polymorphisms at both positions after 20 transfers in the presence of AZC (Table 5), indicating that no virus was dominant in the population at this point. This result represents a clear delay relative to the fixation of both mutations in only 5 transfers when the site-directed mutants competed with the wild type virus.

We also analyzed the behaviour of the site-directed double mutants, Qβ_A1746U+A2982G_ and Qβ_A1746U+U3582C_ in competition experiments with the single mutant Qβ_A1746U_. In both cases the double mutant completely displaced the single mutant after 5 transfers in the presence of AZC (Table 5). However, similar competition experiments carried out by mixing the two double mutants or the two double mutants together with the single mutant Qβ_A1746U_, showed that no virus became dominant after 20 transfers in the presence of AZC (Table 5). This result reinforces the view that the spreading of the genomes containing substitution A2982G interferes with the spreading of the genomes carrying U3582C.

## Discussion

In this work we analyze how beneficial mutations spread in a virus population when replication takes place at increased error rate. To carry out this study we have chosen an RNA virus, the bacteriophage Qβ, which as a consequence of its high error rate [40,41] constitutes extremely heterogeneous populations composed by a complex mutant spectrum [42]. The presence of AZC in the growing medium increased the mutation frequency in viable viruses about an order of magnitude relative to that observed in virus populations evolved in the absence of AZC [31]. The mutation excess (Table 1 and Additional file 1) probably distributes among deleterious, beneficial and neutral mutations, although the much higher frequency of errors having a negative effect on fitness suggests that many of them must be deleterious. Thus, it is expected that beneficial mutations generated in the presence of AZC arise in unfavourable genomic contexts, which can reduce their selective advantages [14-16]. In addition, when the error rate is high enough several beneficial mutations can expand simultaneously, which constitutes another important factor delaying their spreading in the population [12,13]. Thus, although replication at high error rate provides important adaptive advantages to RNA viruses [43,44], it is also true that when subjected to increased mutagenesis, they are frequently pushed to the edge of extinction [45-47], as a result of both the increase in the number of deleterious mutations and the difficulties for the expansion of the beneficial ones.

The analysis of the consensus sequences and the mutant spectra of the AZC-evolved populations showed the fixation of two mutations and the presence of a number of polymorphisms (Table 1) higher than that found during the evolution of the same virus in the absence of AZC (see the two first subsections of Results). It should also be noted that, given the relatively low number of genomes analyzed in each population, the frequency of polymorphic mutations could be even higher than we have reported. Adaptation of bacteriophage MS2 to different selective pressures in the absence of mutagenic conditions also led to the presence of polymorphisms, although to a lower extent to that reported in the current study [26,29]. The findings obtained in studies performed with DNA viruses, which replicate with lower error rate than RNA viruses, are more difficult to compare to our results [30]. Nevertheless, evolution of bacteriophage T7 in the presence of a mutagen also showed a dramatic increase in the number of polymorphic substitutions [48]. Therefore, the high frequency of polymorphisms observed during the evolution of bacteriophage Qβ in the presence of AZC seems to be due, at least in part, to the increase of the error rate.

The high population sizes used for the transmission of the virus (above 10^7^ pfu) together with the absence of clear evidences of hitchhiking suggests that most polymorphic mutations provide a selective advantage, at least in the selective environment provided by the mutagen. The lower values of the ratios ds/dn obtained for the polymorphic mutations than for the non polymorphic ones (Table 3) also supports that selection can be responsible, at least in part, of the high frequency reached by some substitutions. In addition, synonymous changes can also have a fitness effect mediated through *cis* interactions that may be significant in the case of highly compacted genomes, as it is the one of bacteriophage Qβ [49-51]. In contrast to our assumption that most polymorphic mutations are beneficial, there are some studies showing that in natural populations of RNA viruses many high frequency mutations are deleterious, and will be later purged by natural selection [52]. The easiest way for these deleterious mutations to reach high frequency is their linkage with a beneficial mutation. In the absence of clear associations among mutations, this explanation could still be possible if mutations recur frequently as it seems to happen in bacteriophage Qβ (see below).

We have demonstrated that one of the mutations fixed (A2187C) and at least one of the polymorphic substitutions (G3945A) are beneficial in both the presence and the absence of AZC (Table 4) [31], whereas others (A2982G, and U3582C) only provide an advantage in the selective medium, having a fitness cost under standard replication conditions [31]. The results obtained with mutant Qβ_A1746U_ deserve particular attention. This mutant has lower fitness than the virus Qβ_wt_, when they grow independently. However, Qβ_A1746U_ fixes in only 5 transfers when both viruses propagate together in the presence of AZC (Table 4). Fitness is a complex parameter that involves many traits, among which some of the most relevant are the lysis time, the adsorption rate, the burst size, and the stability of the particles outside of the host [53]. It is possible that some of these features influence differently the performance of a virus when it grows isolated or in competition. In good agreement with these considerations Springman et al. [48] reported fitness gains in a population of bacteriophage T7 that, however, showed a clear decline in the burst size.

Our results also showed that the fitness advantages provided by the substitutions assayed were of different magnitude (Tables 4 and 5), which is in disagreement with the proposal of the multiple mutations model which posits a single characteristic value for the coefficient of selection of all beneficial mutations [17,18]. The situation becomes more complex due to mutations whose effects are context dependent. This is the case of substitutions A2982G and U3582C which were beneficial when present in separate genomes, and, however, were lethal or highly deleterious when present in the same genome, providing a clear example of antagonistic epistasis. Another substitution whose effect varied depending on the mutational context and on the competitor genomes was U3989C. This substitution behaved as deleterious in both the presence and the absence of AZC when it was present in the mutational context of the wild type virus (Table 4), and, however, it could be propagated when it was present in the mutational context of substitution A1746U (Figure 2b). These findings support the notion that epistatic interactions among mutations are very frequent in RNA viruses [6,54-58] and can influence the adaptive trajectories followed and the intensity of interference. The increase of the error rate may exacerbate the frequency of this type of interactions, which are not included in any of the current models developed to explain the fixation of beneficial mutations in asexual populations.

The fixation of substitution A1746U was not delayed by the presence in different genomes of either A2982G or U3582C (Table 5). As a consequence, this substitution was probably little affected by interference and could get fixed in population Qβ-AZC(t40). By contrast, substitutions A2982G and U3582C that fixed in 5 transfers when they competed with the wild type virus remained as polymorphisms during at least 20 transfers when they competed with each other (Table 5). A similar competition could be established among the different mutants present in the populations analyzed in this work, supporting that interference among mutations is one of the reasons underlying the sustained presence of polymorphisms.

A phylogenetic analysis carried out with the complete set of genomes obtained at different stages of the evolutionary series allowed us to group them into several clusters that represent independent evolutionary lines that may compete among them (Figure 3). In addition to the mutations representatives of each line, genomes can also contain different mutations among those represented at high frequency, and others that are exclusive of each virus. These mutations can modify the fitness of the genomes where they appear, adding further complexity to the process of interference, and making it difficult to fix any beneficial mutation and the emergence of a defined consensus sequence. The generation of new beneficial mutations in genomes carrying others still not fixed, together with the different magnitude of the effects of beneficial mutations, is more in line with the model of complete interference [21] than with the models of clonal interference [13,14] or multiple mutations [17,18].

Another important finding is that most polymorphic mutations were present in different genomic contexts, suggesting that they were generated repeated times. Results obtained in two adaptation experiments carried out with the bacteriophage MS2 also showed a high number of beneficial mutations in different mutational contexts [26,29]. A theoretical analysis demonstrated that this repeated presence of mutations was more probably due to their generation multiple times than to recombination [26]. The low capability of Qβ replicase to switch between templates does not allow for homologous recombination to be observed in most of the systems assayed [59], as it would be masked by the much higher frequency of punctual mutations [60]. Therefore, we think that the presence of polymorphic mutations in different mutational contexts is more likely due to a high recurrence of punctual mutations than to recombination. This fact would allow combining several beneficial mutations in the same genome, alleviating in this way the costs of interference [61]. However, our results showing that two of the mutations having high selective value in the presence of AZC (A2982G and U3582C) cannot coexist in the same genome suggest that the fitness landscape for bacteriophage Qβ is highly rugged, with the existence of several local maxima where the virus could be trapped without reaching the best adaptive solution.

The high incidence of recurrent mutations could also account for the few hitchhiking mutations found in this study. Given the high mutation frequencies of the bacteriophage Qβ populations evolved in the presence of AZC, it would seem reasonable that almost each genome with a beneficial mutation in the process to fixation will also carry a set of hitchhiking mutations. However, the repeated occurrence of the beneficial mutations on different backgrounds also means that no single set of accompanying mutations gets fixed. A similar result was reported for bacteriophage MS2 adapting to cold temperatures [29], and was also supported by theoretical calculations [62].

Although at a first glance the interference among mutations can be seen as a negative feature that delays adaptation, there are also positive consequences that deserve to be pointed out. Maybe one of the most relevant is the coexistence in the population of multiple adaptive possibilities that can provide selective advantages by different molecular mechanisms. Population bottlenecks occurring during the propagation of the virus can lead to the fixation of different adaptive solutions. In this way, interference could be seen as a positive factor contributing to the diversification of populations, and permitting the exploration of the different local maxima that exist in rugged fitness landscapes, such as those described for RNA viruses [63,64].

Interference among mutations can also play a relevant role in the extinction of viruses through lethal mutagenesis, a new antiviral strategy that derives from theoretical considerations [65-67] and that consists in the treatment of virus infections through the artificial increase of the virus error rate [46,47,68,69]. Our results show that virus replication under mutagenic conditions can lead to the simultaneous presence in the mutant spectrum of multiple mutations conferring different advantages in the presence of the mutagen. The fixation of these mutations in particular individuals upon transmission of the virus through population bottlenecks, as indicated above, can lead to the co-circulation of viruses differing in their adaptive properties, jeopardizing in this way the efficacy of further treatments. Given the high error rates of RNA viruses, similar situations could also occur during the treatment with some replication inhibitors. Therefore, it is expected that future research on the evolutionary consequences of the interference among mutations also provides significant benefits to clinic and epidemiologic virology.

## Conclusions

Evolution of bacteriophage Qβ at artificially increased error rate by means of the use of a mutagenic nucleoside analogue leads to the prolonged permanence of multiple polymorphisms which could be detected in both the consensus sequences and the mutant spectra.

Polymorphic mutations have different effects on fitness. Some of them provide selective advantages in both the presence and the absence of AZC, whereas others only are beneficial under selective conditions, having a fitness cost under standard replication conditions. Epistatic interactions also play a role in deciding whether a particular mutation will reach high frequency or not.

Polymorphic mutations distribute into multiple evolutionary lines that compete among them making it difficult the emergence of a defined consensus sequence. Each evolutionary line can provide a selective advantage by a different molecular mechanism leading to the coexistence of multiple adaptive pathways in the same population.

Antagonistic epistasis determines that two of the replicase mutations providing selective advantages in the presence of AZC cannot associate in the same genome. As a consequence, genomes carrying each of these mutations spread simultaneously delaying the fixation of any of them.

The variety of mutational contexts in which most of the polymorphic mutations have been detected indicates that beneficial mutation recur frequently in bacteriophage Qβ. This circumstance could alleviate the disadvantages caused by interference. However, the fact that some of the adaptive substitutions cannot be combined in the same genome limits the potential benefits of the repeated generation of beneficial mutations.

The fixation of mutations in bacteriophage Qβ evolved in the presence of AZC is better approached by the model of complete interference than by the models of clonal interference or multiple mutations. However, the presence of epistatic interactions and the high frequency of recurrent mutations contribute to generate a highly complex interference dynamics, which would require further improvements of the theoretical models to be successfully described.

## Competing interests

The authors declare that they do not have competing interests.

## Authors’ contributions

MA and LC contributed equally to this paper. EL designed the study. MA and LC carried out the experiments. EL, MA, and LC interpreted the results. EL wrote the paper. All the authors read and approved the final manuscript.

## Supplementary Material

Additional file 1 **Additional substitutions to those represented at high frequency in the virus genomes analyzed.** Description: It contains all the substitutions that are not represented at high frequency in the mutant spectra of the virus populations analyzed. Genomes correspond to those indicated in Table 1. Click here for file

## References

[B1] Muller HJ Some genetic aspects of sex Am Nat 1932 68 118 138

[B2] Muller HJ The relation of recombination to mutational advance Mutat Res 1964 106 2 9 14195748 10.1016/0027-5107(64)90047-8

[B3] Atwood KC Schneider LK Ryan FJ Periodic selection in Escherichia coli Proc Natl Acad Sci USA 1951 37 146 155 10.1073/pnas.37.3.146 14808170 PMC1063322

[B4] Papadopoulos D Schneider D Meier-Eiss J Arber W Lenski RE Blot M Genomic evolution during a 10,000-generation experiment with bacteria Proc Natl Acad Sci USA 1999 96 3807 3812 10.1073/pnas.96.7.3807 10097119 PMC22376

[B5] Wichman HA Badgett MR Scott LA Boulianne CM Bull JJ Different trajectories of parallel evolution during viral adaptation Science 1999 285 422 424 10.1126/science.285.5426.422 10411508

[B6] Holder KK Bull JJ Profiles of adaptation in two similar viruses Genetics 2001 159 1393 1404 11779783 10.1093/genetics/159.4.1393 PMC1461900

[B7] Rozen DE de Visser JA Gerrish PJ Fitness effects of fixed beneficial mutations in microbial populations Curr Biol 2002 12 1040 1045 10.1016/S0960-9822(02)00896-5 12123580

[B8] Shaver AC Dombrowski PG Sweeney JY Treis T Zappala RM Sniegowski PD Fitness evolution and the rise of mutator alleles in experimental Escherichia coli populations Genetics 2002 162 557 566 12399371 10.1093/genetics/162.2.557 PMC1462288

[B9] de Visser JA Rozen DE Clonal interference and the periodic selection of new beneficial mutations in Escherichia coli Genetics 2006 172 2093 2100 16489229 10.1534/genetics.105.052373 PMC1456385

[B10] Lang GI Botstein D Desai MM Genetic variation and the fate of beneficial mutations in asexual populations Genetics 2011 188 647 661 10.1534/genetics.111.128942 21546542 PMC3176544

[B11] de Visser JA Rozen DE Limits to adaptation in asexual populations J Evol Biol 2005 18 779 788 10.1111/j.1420-9101.2005.00879.x 16033549

[B12] Sniegowski PD Gerrish PJ Beneficial mutations and the dynamics of adaptation in asexual populations Philos Trans R Soc Lond B Biol Sci 2010 365 1255 1263 10.1098/rstb.2009.0290 20308101 PMC2871819

[B13] Gerrish PJ Lenski RE The fate of competing beneficial mutations in an asexual population Genetica 1998 102-103 127 144 9720276

[B14] Wilke CO The speed of adaptation in large asexual populations Genetics 2004 167 2045 2053 10.1534/genetics.104.027136 15342539 PMC1470994

[B15] Orr HA The rate of adaptation in asexuals Genetics 2000 155 961 968 10835413 10.1093/genetics/155.2.961 PMC1461099

[B16] Orr HA The distribution of fitness effects among beneficial mutations Genetics 2003 163 1519 1526 12702694 10.1093/genetics/163.4.1519 PMC1462510

[B17] Desai M Fisher DS Beneficial mutation-selection balance and the effect of linkage on positive selection Genetics 2007 176 1759 1798 10.1534/genetics.106.067678 17483432 PMC1931526

[B18] Desai M Fisher DS Murray AW The speed of evolution and maintenance of variation in asexual populations Curr Biol 2007 17 385 394 10.1016/j.cub.2007.01.072 17331728 PMC2987722

[B19] Brunet E Rouzine IM Wilke CO The stochastic edge in adaptive evolution Genetics 2008 179 603 620 10.1534/genetics.107.079319 18493075 PMC2390637

[B20] Hegreness M Shoresh N Hartl D Kishony R An equivalence principle for the incorporation of favorable mutations in asexual populations Science 2006 17 311 1615 1617 16543462 10.1126/science.1122469

[B21] Park SC Krug J Clonal interference in large populations Proc Natl Acad Sci USA 2007 104 18135 18140 10.1073/pnas.0705778104 17984061 PMC2084309

[B22] de Visser JA Zeyl CW Gerrish PJ Blanchard JL Lenski RE Diminishing returns from mutation supply rate in asexual populations Science 1999 283 404 406 10.1126/science.283.5400.404 9888858

[B23] Barrick JE Lenski RE Genome-wide mutational diversity in an evolving population of Escherichia coli Cold Spring Harb Symp Quant Biol 2009 74 119 29 10.1101/sqb.2009.74.018 19776167 PMC2890043

[B24] Miralles R Gerrish PJ Moya A Elena SF Clonal interference and the evolution of RNA viruses Science 1999 285 1745 1747 10.1126/science.285.5434.1745 10481012

[B25] Kao KC Sherlock G Molecular characterization of clonal interference during adaptive evolution in asexual populations of Saccharomyces cerevisiae Nat Genet 2008 40 1499 1504 10.1038/ng.280 19029899 PMC2596280

[B26] Bollback JP Huelsenbeck JP Clonal interference is alleviated by high mutation rates in large populations Mol Biol Evol 2007 24 1397 1406 10.1093/molbev/msm056 17379621

[B27] Gresham D Desai MM Tucker CM Jenq HT Pai DA Ward A DeSevo CG Botstein D Dunham MJ The repertoire and dynamics of evolutionary adaptations to controlled nutrient-limited environments in yeast PLoS Genet 2008 4 12 e1000303 10.1371/journal.pgen.1000303 19079573 PMC2586090

[B28] Barrick JE Yu DS Yoon SH Jeong H Oh TK Schneider D Lenski RE Kim JF Genome evolution and adaptation in a long-term experiment with Escherichia coli Nature 2009 461 1243 1247 10.1038/nature08480 19838166

[B29] Betancourt AJ Genomewide patterns of substitution in adaptively evolving populations of the RNA bacteriophage MS2 Genetics 2009 181 1535 1544 10.1534/genetics.107.085837 19189959 PMC2666518

[B30] Miller CR Joyce P Wichman HA Mutational effects and population dynamics during viral adaptation challenge current models Genetics 2011 187 185 202 10.1534/genetics.110.121400 21041559 PMC3018297

[B31] Arribas M Cabanillas L Lázaro E Identification of mutations conferring 5-azacytidine resistance in bacteriophage Qβ Virology 2011 417 343 352 10.1016/j.virol.2011.06.016 21757215

[B32] Cases-González C Arribas M Domingo E Lázaro E Beneficial effects of population bottlenecks in an RNA virus evolving at increased error rate J Mol Biol 2008 384 1120 1129 10.1016/j.jmb.2008.10.014 18951905

[B33] Barrera I Schuppli D Sogo JM Weber H Different mechanisms of recognition of bacteriophage Qβ plus and minus strand RNAs by Qβ replicase J Mol Biol 1993 232 512 521 10.1006/jmbi.1993.1407 8345521

[B34] The HIV databaseshttp://www.hiv.lanl.gov

[B35] Korber B Rodrigo AG, Learn GH HIV Signature and Sequence Variation Analysis Computational Analysis of HIV Molecular Sequences 2000 Dordrecht, Netherlands: Kluwer Academic Publishers 55 72

[B36] Lynch M Gabriel W Mutational load and the survival of small populations Evolution 1990 44 1725 1737 10.2307/2409502 28567811

[B37] Lázaro E Escarmís C Pérez-Mercader J Manrubia SC Domingo E Resistance of virus to extinction on bottleneck passages: study of a decaying and fluctuating pattern of fitness loss Proc Natl Acad Sci USA 2003 100 10830 10835 10.1073/pnas.1332668100 12960384 PMC196888

[B38] Escarmís C Lázaro E Manrubia SC Population bottlenecks in quasispecies dynamics Curr Top Microbiol Immunol 2006 299 141 70 10.1007/3-540-26397-7_5 16568898

[B39] Guindon S Dufayard JF Lefort V Anisimova M Hordijk W Gascuel O New algorithms and methods to estimate maximum-likelihood phylogenies: assessing the performance of PhyML 3.0 Syst Biol 2010 59 307 321 10.1093/sysbio/syq010 20525638

[B40] Drake JW Rates of spontaneous mutation among RNA viruses Proc Natl Acad Sci USA 1993 90 4171 4175 10.1073/pnas.90.9.4171 8387212 PMC46468

[B41] Drake JW Charlesworth B Charlesworth D Crow JF Rates of spontaneous mutation Genetics 1998 148 1667 1686 9560386 10.1093/genetics/148.4.1667 PMC1460098

[B42] Domingo E Sabo D Taniguchi T Weissmann C Nucleotide sequence heterogeneity of an RNA phage population Cell 1978 13 735 744 10.1016/0092-8674(78)90223-4 657273

[B43] Pfeiffer JK Kirkegaard K Increased fidelity reduces poliovirus fitness and virulence under selective pressure in mice PLoS Pathog 2005 1 2 e11 10.1371/journal.ppat.0010011 16220146 PMC1250929

[B44] Vignuzzi M Stone JK Arnold JJ Cameron CE Andino R Quasispecies diversity determines pathogenesis through cooperative interactions in a viral population Nature 2006 439 344 348 10.1038/nature04388 16327776 PMC1569948

[B45] Holland JJ Domingo E de la Torre JC Steinhauer DA Mutation frequencies at defined single codon sites in vesicular stomatitis virus and poliovirus can be increased only slightly by chemical mutagenesis J Virol 1990 64 3960 3962 1695258 10.1128/jvi.64.8.3960-3962.1990 PMC249691

[B46] Loeb LA Essigmann JM Kazazi F Zhang J Rose KD Mullins JI Lethal mutagenesis of HIV with mutagenic nucleoside analogs Proc Natl Acad Sci USA 1999 96 1492 1497 10.1073/pnas.96.4.1492 9990051 PMC15492

[B47] Domingo E Virus entry into error catastrophe as a new antiviral strategy Virus Res 2005 107 115 228 10.1016/j.virusres.2004.11.001

[B48] Springman R Keller T Molineux IJ Bull JJ Evolution at a high imposed mutation rate: adaptation obscures the load in phage T7 Genetics 2010 184 221 232 10.1534/genetics.109.108803 19858285 PMC2815918

[B49] Mills DR Priano C Merz P Binderow BD Qβ RNA bacteriophage: mapping cis-acting elements within an RNA genome J Virol 1990 64 3872 3881 2196383 10.1128/jvi.64.8.3872-3881.1990 PMC249683

[B50] Klovins J Berzins V van Duin J A long-range interaction in Qbeta RNA that bridges the thousand nucleotides between the M-site and the 3′ end is required for replication RNA 1998 4 948 957 10.1017/S1355838298980177 9701286 PMC1369672

[B51] Klovins J van Duin J A long-range pseudoknot in Qbeta RNA is essential for replication J Mol Biol 1999 294 875 884 10.1006/jmbi.1999.3274 10588893

[B52] Pybus OG Rambaut A Belshaw R Freckleton RP Drummond AJ Holmes EC Phylogenetic evidence for deleterious mutation load in RNA viruses and its contribution to viral evolution Mol Biol Evol 2007 24 845 852 17218639 10.1093/molbev/msm001

[B53] Shao Y Wang IN Bacteriophage adsorption rate and optimal lysis time Genetics 2008 180 471 482 10.1534/genetics.108.090100 18757924 PMC2535697

[B54] Sanjuán R Moya A Elena SF The contribution of epistasis to the architecture of fitness in an RNA virus Proc Natl Acad Sci USA 2004 101 15376 15379 10.1073/pnas.0404125101 15492220 PMC524436

[B55] Sanjuán R Cuevas JM Moya A Elena SF Epistasis and the adaptability of an RNA virus Genetics 2005 170 1001 1008 10.1534/genetics.105.040741 15879507 PMC1451175

[B56] Sanjuán R Elena SF Epistasis correlates to genomic complexity Proc Natl Acad Sci USA 2006 103 14402 14405 10.1073/pnas.0604543103 16983079 PMC1599975

[B57] Weinreich DM Watson RA Chao L Perspective: Sign epistasis and genetic constraint on evolutionary trajectories Evolution 2005 59 1165 1174 16050094

[B58] Betancourt AJ Lack of evidence for sign epistasis between beneficial mutations in an RNA bacteriophage J Mol Evol 2010 71 437 443 10.1007/s00239-010-9397-0 20938652

[B59] Chetverin AB Chetverina HV Demidenko AA Ugarov VI Nonhomologous RNA recombination in a cell-free system: evidence for a transesterification mechanism guided by secondary structure Cell 1997 88 503 513 10.1016/S0092-8674(00)81890-5 9038341 PMC7173214

[B60] Palasingam K Shaklee PN Reversion of Q beta RNA phage mutants by homologous RNA recombination J Virol 1992 66 2435 2442 1548770 10.1128/jvi.66.4.2435-2442.1992 PMC289039

[B61] Kim Y Orr HA Adaptation in sexuals vs. asexuals: clonal interference and the Fisher-Muller model Genetics 2005 171 1377 1386 10.1534/genetics.105.045252 16020775 PMC1456838

[B62] Charlesworth B Morgan MT Charlesworth D The effect of deleterious mutations on neutral molecular variation Genetics 1993 134 1289 1303 8375663 10.1093/genetics/134.4.1289 PMC1205596

[B63] Elena SF Sanjuán R RNA viruses as complex adaptive systems Biosystems 2005 81 31 41 10.1016/j.biosystems.2005.02.001 15917126

[B64] Lalić J Elena SF Magnitude and sign epistasis among deleterious mutations in a positive-sense plant RNA virus Heredity 2012 109 71 7 10.1038/hdy.2012.15 22491062 PMC3400743

[B65] Eigen M Schuster P The hypercycle. A principle of natural self-organization 1979 Berlin: Springer 10.1007/BF00450633 593400

[B66] Nowak M Schuster P Error thresholds of replication in finite populations mutation frequencies and the onset of Muller's ratchet J Theor Biol 1989 137 375 395 10.1016/S0022-5193(89)80036-0 2626057

[B67] Biebricher CK Eigen M The error threshold Virus Res 2005 107 117 127 10.1016/j.virusres.2004.11.002 15649558

[B68] Eigen M Error catastrophe and antiviral strategy Proc Natl Acad Sci USA 2002 99 13374 13376 10.1073/pnas.212514799 12370416 PMC129678

[B69] Lázaro E RNA Viruses: Control, Mutagenesis and Extinction eLS Chichester: John Wiley & Sons, Ltd 10.1002/9780470015902.a0023276

