# Supplementary material for: Evolution at increased error rate leads to the coexistence of multiple adaptive pathways in an RNA virus
Source: BMC Evol Biol. 2013 Jan 16;13:11. doi: 10.1186/1471-2148-13-11 (PMC3556134; doi:10.1186/1471-2148-13-11)
Supplement: Additional file 1 — Additional substitutions to those represented at high frequency in the virus genomes analyzed. Description: It contains all the substitutions that are not represented at high frequency in the mutant spectra of the virus populations analyzed. Genomes correspond to those indicated in Table 1. [file 1471-2148-13-11-S1.pdf]

Additional substitutions to those represented at high frequency in the virus genomes analyzed.

Virus clone[1]	Substitutions
Population Qβ-AZC(t60)	
C_1	U1583C, U3020C
C_2	C1625U, U2129C, U3044C
C_3	C1625G, U1652C, U2213C
C_4	C1625G, U2966C, A3157G, U3516C
C_5	A1778G, A2508G, U3011C, U3170G, U3212C, A3566G
C_6	A1890G, U1964C, U2230C, U2460C, A2508G, U2608C, U2740C, A2948G, U3419C, U3484C
C_7	U1796C, U2061C, U2509C, U2776C, A2915G, U3056C, C3455G, U3899C
C_8	A2094C, U2495C, A3260G, A3504C
C_9	U1628C, U2009C, U2426C, U2467C, U2776C, U3008C, A3504C
C_{10}	U2276G, U2367A, G2524A, A2556G, A2590G, U3029C, C3193G, U3569C, U3734C
Population Qβ-AZC(t70)	
C_1	U2105C, U2900C, U3068C,
C_2	C1517G, U2123C, U2951C, U3038C, U3287C, U3314C,
C_3	C1955G, U2009C, A2119G, U2276G, U3867C,
C_4	U1601C, C1625G, U1703C, U2105C, A2160G, C2210A, U2276C, U2425C, U2495C, U2558C, U3011C, C3158G, C3233G

C_5	A1574G, A1898G, U2060C, U2425C, U2558C, U2776C, A2949G, C3158G, A3268G, U3314C, U3737C
C_6	U1628C, A2657G, A2943G, U3122C, U3356A, U3755C, U3761C, A3824G
C_7	U1886C, C1962G, A2656G, A2810C, U3356C, A3504C
C_8	A1890G, U2291C, U2509C, U2777C, U2954C, G3994C
C_9	A1867G, G2214A, G2254U, G2256U, C2371U, U2495C, U3438G, C3659U, U3956C
C_{10}	U1796G, U2016C, A2412G, U2509C, U2776C, U3020C, C3455G, C3879G

Population Qβ-AZC(t90)

C_1	A1827G, A2293G, U2477G, U2510C, U3212C, C3344G, U3590C
C_2	C1550U, U2042C, A2161G, G3053A, U3219C, U3245G, U3314C, C3545A, U3791C, U3900C
C_3	U1505G, U1965C, A2422G, U2459C, U2684C, A2810G, A2831G, A2948G, C3074G, U3101G, U3788C
C_4	U2016C, U2609C, C2799G, A2948U, U3245C, U3266G, A3268G, U3380C, U3750C
C_5	A1957G, U2936C, U2966C, C3205G, C3302U
C_6	A1867G, A1893G, A2366G, A2831G, C3158A, C3206U, U3362C
C_7	A2119G, A2773G, U2825C, A3179G, U3473C, U3755C
C_8	U1954C, U3245C
C_9	G1826U, U1934C, A1956G, C1962G, A2010U, U2291C, U2459C, U2663C, U2785C, A2810U, U3056C, U3314C, U3359C, U3419C, U3684C, U3788C, A3813G, U3878C, U3902C
C_{10}	A2094G, U2291C, U2495C, U2510C, A2657G, U2777C
C_{11}	U1505G, U1811C, U1964C, U2099C, U2105C, U2420C, U2425C, C3158G, A3941G

C_{12}	A1867G, U1886C, C1962G, U1964C, U2300C, U2379C, C3233G, A3504C, U3684C
C_{13}	U1583C, U1682C, U1721C, U1811C, U2213C, C2929G, U3431C, A3506U
C_{14}	A1891G, U2675C, A2949G, C3158G, A3504C, U3605C, C3659U, A3911G
C_{15}	U2129C, U2813C, U2972C, U3412C

[1]Virus clones correspond to those indicated in Table 1.
